# The Computational Boundary of a “Self”: Developmental Bioelectricity Drives Multicellularity and Scale-Free Cognition

**DOI:** 10.3389/fpsyg.2019.02688

**Published:** 2019-12-13

**Authors:** Michael Levin

**Affiliations:** ^1^Allen Discovery Center at Tufts University, Medford, MA, United States; ^2^Wyss Institute for Biologically Inspired Engineering, Harvard University, Boston, MA, United States

**Keywords:** development, bioelectricity, gap junctions, primitive cognition, active inference

## Abstract

All epistemic agents physically consist of parts that must somehow comprise an integrated cognitive self. Biological individuals consist of subunits (organs, cells, and molecular networks) that are themselves complex and competent in their own native contexts. How do coherent biological Individuals result from the activity of smaller sub-agents? To understand the evolution and function of metazoan creatures’ bodies and minds, it is essential to conceptually explore the origin of multicellularity and the scaling of the basal cognition of individual cells into a coherent larger organism. In this article, I synthesize ideas in cognitive science, evolutionary biology, and developmental physiology toward a hypothesis about the origin of Individuality: “Scale-Free Cognition.” I propose a fundamental definition of an Individual based on the ability to pursue goals at an appropriate level of scale and organization and suggest a formalism for defining and comparing the cognitive capacities of highly diverse types of agents. Any Self is demarcated by a computational surface – the spatio-temporal boundary of events that it can measure, model, and try to affect. This surface sets a functional boundary - a cognitive “light cone” which defines the scale and limits of its cognition. I hypothesize that higher level goal-directed activity and agency, resulting in larger cognitive boundaries, evolve from the primal homeostatic drive of living things to reduce stress – the difference between current conditions and life-optimal conditions. The mechanisms of developmental bioelectricity - the ability of all cells to form electrical networks that process information - suggest a plausible set of gradual evolutionary steps that naturally lead from physiological homeostasis in single cells to memory, prediction, and ultimately complex cognitive agents, *via* scale-up of the basic drive of infotaxis. Recent data on the molecular mechanisms of pre-neural bioelectricity suggest a model of how increasingly sophisticated cognitive functions emerge smoothly from cell-cell communication used to guide embryogenesis and regeneration. This set of hypotheses provides a novel perspective on numerous phenomena, such as cancer, and makes several unique, testable predictions for interdisciplinary research that have implications not only for evolutionary developmental biology but also for biomedicine and perhaps artificial intelligence and exobiology.

## Introduction: Deep Problems With Something Fundamental in Common

Why did some competent unicellular organisms join together to form complex bodies, and how do they cooperate during highly robust embryogenesis and regeneration of anatomical structures? Why does this process break down during carcinogenic defection from the body plan? How can we best understand and control biological systems that consist of numerous nested levels of organization, such as bacteria and biofilms, which can functionally interact with the host’s cells, tissues, and organs? How are lower level (molecular and cellular) activities harnessed toward adaptive system-level outcomes during regulative development and adaptation to novel stressors? What is the relationship between the ability of cells to implement invariant organ-level morphogenetic goal states and the purposive activity of brains? What dynamics enable the scaling of cognitive capacities from the simple memory functions found in bacteria to those of sophisticated minds?

All these fundamental biological problems have one thing in common: the need to understand and formalize what a coherent Individual or Agent is, in a way that is compatible with a gradual co-evolution of minds and bodies (Tarnita et al., 2013). While significant work has addressed this topic from an evolutionary perspective, I suggest a different and complementary view called Scale-Free Cognition, synthesizing ideas from theories of computation and control to identify common information-processing events occurring at multiple levels of organization. I propose a semi-quantitative metric, based on the spatio-temporal boundaries of events that systems measure and try to control, that can be used to define and compare the cognitive boundaries for highly diverse types of agents (which could be biological, exo-biological, or artificial). Ideas from the fields of proto-cognition, developmental biophysics, and information theory offer a novel lens with which to understand the evolution, development, physiology, and behavior of a wide range of living systems. Focusing on information processing and decision-making enables a unifying conception of goal-directedness in biological systems, which naturally scales from simple homeostatic pathways to complex cognition *via* evolutionarily ancient physiological mechanisms of cell-cell communication. Here, I illustrate these hypotheses from the perspective of developmental bioelectricity, which evolution has robustly exploited for cognitive scaling; however, the same general scheme applies to any similar mechanism, whether chemical, physical, or other.

**Figure 1:**
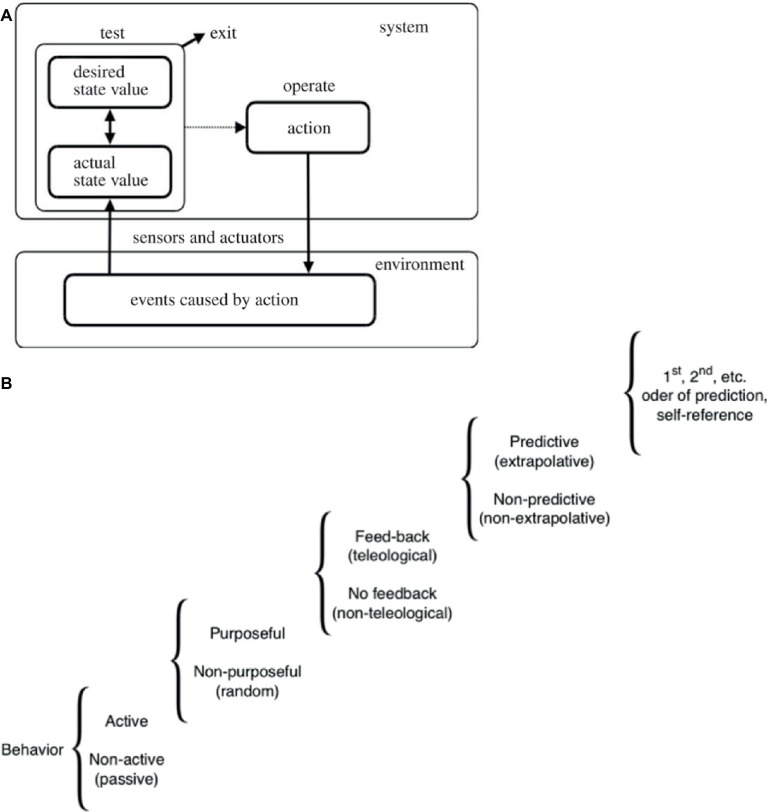
A continuum of cognitive powers. **(A)** A “TOTE” (test, operate, test, exit) loop schematizing a basic homeostatic cycle. This system’s unique architecture applies to many possible types of sensors and effectors at different scales of organization. By continuously taking action to minimize the distance (difference, error) between the current state of affairs and a set point describing a different (possible future) state of affairs, it enables a system to pursue goals despite perturbations from the outside world and intrinsic noise. Taken with permission from Pezzulo and Levin (2016). **(B)** This scale shows how different types of activity in systems can be ranked according to their degree of purposiveness. Many different types of cognition, from the simplest mechanisms to complex thought, illustrate that “cognitive system” is not a binary designation but rather a step on a continuum of computational capacity along which very diverse systems can be placed. Modified after Rosenblueth et al. (1943).

These ideas are explored here from the perspective of the following three core assumptions. First is a commitment to evolution: every capacity has a natural history and emerged from simpler variants. Closely related is the idea that cognition, like “organism status” (Child, 1915; Queller and Strassmann, 2009), is not a binary capacity that exists in higher organisms only (Figure 1). Thus, it is fundamentally incorrect to view functional capacities such as memory, prediction, goal-directedness, etc. as entirely new features appropriate only to advanced forms of life, or to view descriptions of cognitive capacities in primitive or aneural organisms as category mistakes (Baluška and Levin, 2016). Indeed, failure to appreciate cognitive processes in a system that would have otherwise improved prediction and control is a “neganthropomorphic fallacy” – a type 1 error that is as bad for empirical research as is profligate anthropomorphic reasoning. This is a kind of “intentional stance” (Dennett, 1987) approach, generalized beyond brains and behavior. Thus, here it is assumed that there is no such thing as magical “true cognition” that is the province of humans, without a smooth history of precursor capacities in simpler forms, stretching back to the base of the tree of life (Dennett, 2017; Ginsburg et al., 2019). Second, it is assumed that all metaphors are to be judged by their utility in driving scientific progress and that there is not a binary categorization of scientific pictures which should be taken literally or not, which can be decided *a priori*. Thus, whether a way of thinking about a system is correct or mere metonymy is to be determined by whether the specific metaphor gives rise to new, robust research programs – it is an empirical question to be answered in time, based on whether a given metaphor improves prediction and control in novel cases at the bench, compared to other existing metaphors. Third, much of the discussion centers around “goals”, related to teleology, a hotly debated topic (Nagel, 1979; Ruse, 1989; Teufel, 2011; McShea, 2012, 2013, 2016; Kolchinsky and Wolpert, 2018): here goal-directedness is taken in the non-magical, cybernetic, engineering, and control theory sense, of a feedback system that operates to maximize some specific state of affairs (which can be modeled as a dynamical system with attractors in its state space). Finally, except for a few brief remarks at the end, no claims about consciousness (first person experience or a sense of self as qualia) are made: all of the examples concern functional, third-person, objective capacities, computations, and behaviors.

## What is a Self? Defining “Individuals”

“Of course there is no question that a tree or an elephant is one individual, and we have a very clear mental picture of what this means, for we ourselves are individuals. But there are lower forms in the borderland between one-celled organisms and multicellular organisms that are more bothersome in this respect.”

–J. T. Bonner, 1950

Neuroscientists (and philosophers of mind) have long wrestled with the question of how many can add up to one – a “conceptual self that is composed of interacting neural regions” with centralized agency and planning (Murray et al., 2015). But this is not just a key question for neurobiology: as will be seen below, important aspects of biomedicine also hinge on the ability to identify higher-order control structures composed of cells or pathways. Epistemology requires a subject (whoever it is that does or does not know something), and this is critical not only for human philosophers but also for many animals. Survival depends on evolving finely tuned agency detectors that allow them to parse their world into agents that pursue recognizable goals and can be interacted with in a way different than with their parts (Mar et al., 2007).

Exobiologists may at some point be confronted with the task of understanding what aspect of an alien ecosystem constitutes some sort of coherent biological Individual. This is likely to be a very non-trivial task, as even life on Earth presents a significant challenge in the cases of metazoan microbiomes and colonial/symbiotic organisms (Daniels et al., 2016). In addition to evolved/natural Agents, the field of artificial intelligence has (or soon will) give rise to numerous constructs that may represent kinds of Selves. The goal then is not to attempt to draw a sharp line but to understand the factors that go into empirically useful ways to demarcate Individuals in a given context.

The question of defining an “organism” has been long discussed in biology (Huxley, 1852; Loeb, 1916, 1937; Sober, 1991; Maynard Smith and Szathmáry, 1995; West et al., 2015). Much work has been done on defining compound Individuals, from perspectives of evolution (the forces which drive long-term changes in the parts and how they relate), game theory (competition between and within the parts), thermodynamics and dissipative systems, systems theory, and even immunology (Prigogine, 1980; Rosen, 1985; Pradeu and Carosella, 2006; Strassmann and Queller, 2010; Pradeu, 2012, 2016; Godfrey-Smith, 2016, 2017). Below, I first present some background on aspects of flexible decision-making at different levels of biological organization and then argue for a definition based on information and goal-directedness (Walker et al., 2016) from a developmental biology perspective. I suggest candidates for proximate mechanisms that drive major transitions and provide a plausible evolutionary story of how primitive homeostasis leads to advanced agency (Man and Damasio, 2019). This perspective is complementary to others, not incompatible with them. There is likely more than one useful definition of what constitutes a cognitive agent, in terms of making predictions and optimizing control policies for the origin and behavior of specific agents in various circumstances. As will be seen below, the information-centered definition of the Self has specific advantages for dealing with nested (multi-scale) structures observed in the biosphere and for understanding the shifting (not fixed) boundaries between Self and environment that can change within the timeline of a single individual (not only on evolutionary timescales).

## Body Patterning and Cognition: A Common Origin

“Our life is shaped by our mind; we become what we think.”

— Gautama Buddha

It is a well-known fact that the biosphere is a set of nesting dolls (Smythies, 2015). Eco-systems consist of groups that are comprised of organisms, which in turn are made of organs composed of tissues, which consist of cells made up of biochemical networks.

Remarkably, flexible and adaptive behavior is found at every level, which provides an important background for thinking about scale-invariant, essential features of Individuals in the broadest sense. Do integrated Selves only exist at the level of “organisms” (bodies), or could they arise and co-exist at multiple levels of organization and be recognized in novel contexts and implementations? In preparation for a proposed definition of Selves as goal-directed computational agents regardless of implementation, it is helpful to begin by considering novel embodiments of capacities usually associated with brains.

Single cells are composite agents that exhibit extremely rich patterning and behaviors and can be divided into even smaller independent units (such as cytoplast fragments with autonomous activity) because of their dynamic cytoskeletal and protein network subsystems (Novák and Bentrup, 1972; Albrecht-Buehler, 1985; Ford, 2017; Siccardi and Adamatzky, 2017; Barvitenko et al., 2018; Graham et al., 2018). These capacities of cells presage their swarm behavior as metazoan organisms (Gregg, 1959). Most features observed in the anatomical control of complex organisms, including differentiation (Sogabe et al., 2019), plasticity (Koch et al., 2017), programmed cell death (Gordeeva et al., 2004), regenerative repair (Morgan, 1901), and “neural” machinery (Tasneem et al., 2005; Liebeskind et al., 2011; Burkhardt, 2015) already existed in ancient, unicellular life forms. Cells such as bacteria and yeast, as well as advanced plants, have been studied for their ability to compute and predict future events using patterns inferred from prior experiences (Saigusa et al., 2008; Pezzulo and Castelfranchi, 2009; Stepp, 2009; Shemesh et al., 2010; Stepp and Turvey, 2010; Mossbridge et al., 2012; Dhar et al., 2013; Goel and Mehta, 2013; Bohm et al., 2016; De Berker et al., 2016; Katz and Springer, 2016; Peters et al., 2017; Wilson et al., 2017; Gagliano et al., 2018; Katz et al., 2018). Such aneural systems have also been used to understand neural function (Koshland, 1983; Morimoto and Koshland, 1991; Sarto-Jackson and Tomaska, 2016). Thus, capacities usually assigned to Individuals with nervous systems, such as integrating spatio-temporal information, memory, and ability to pursue specific outcomes *via* selection from a number of possible behaviors evolved from far older pre-neural origins (Eisenstein, 1975; Boisseau et al., 2016).

The emerging field known as “basal cognition” tracks the evolutionary history of learning and decision-making processes, beginning from the dynamic problem-solving capacities of cellular and subcellular forms (Lyon, 2006, 2015; Ginsburg et al., 2019). Many examples of memory, anticipation, context-dependent decision-making, and learning are exhibited by organisms from yeast and bacteria to plants and somatic cells [reviewed in (Lyon, 2006, 2015; Baluška and Levin, 2016)]. This is even true of subcellular-level components, e.g., gene-regulatory networks can execute similar learning and computational properties as neural networks, as can cytoskeletal networks, cell signaling pathways, reaction-diffusion chemistry, and metabolic networks (Watson et al., 2010; Szabó et al., 2012; Stockwell et al., 2015; Prentice-Mott et al., 2016; Dent, 2017; Stovold and O’Keefe, 2017; Barvitenko et al., 2018; Gabalda-Sagarra et al., 2018; Bulcha et al., 2019). Single cells are very good at managing their morphology, behavior, and physiology as needed for survival, altering their motility, and metabolism in response to, and proactively in, changing environmental conditions. They succeed in exploiting their microenvironment toward optimal reproduction by selecting among numerous possible choices of gene expression patterns and behaviors. While the mechanisms by which unicellular organisms’ ability to accomplish specific adaptive ends is harnessed toward cooperative multicellularity is still poorly understood, one thing is clear: somatic cells did not lose their behavioral plasticity and computational capabilities in becoming part of metazoan swarms (bodies): they scaled them to enable pursuit of larger goals consisting of creation and upkeep of massively complex anatomies (Pezzulo and Levin, 2015).

Metazoan embryos are possible because the progeny of a fertilized egg cell can cooperate to create an invariant, large, complex anatomical structure with very high fidelity. Crucially, this is not simply an emergent result of hardwired processes but a very plastic, context-dependent system that achieves invariant patterning outcomes in an uncertain world. Regulative development (e.g., the two normal bodies resulting when an early embryo is cut in half) reveals that cellular swarms are able to achieve the same desired end-state (a species-specific target morphology) despite drastic perturbations and highly unexpected starting states. For example, when tadpoles are perturbed in the laboratory such that their craniofacial organs are in abnormal positions, they still make largely normal frog faces because eyes, jaws, and other structures move around in un-natural paths and only stop when a “correct frog face configuration” is reached (Vandenberg et al., 2012; Pinet and McLaughlin, 2019). Similarly, salamander tails grafted to the flank slowly remodel into limbs – a structure more compatible with the large-scale anatomical spec of a salamander (Farinella-Ferruzza, 1956). Many animals, such as axolotls, are able to regenerate whole limbs, no matter where they are amputated, and other organs; a key aspect is that the new appendages grow until the precisely correct structures are made – no more and no less. Developing kidneys will form tubules of the same diameter, whether by cell-cell communication of ~10 cells per cross-section in normal settings or by cytoskeletal bending of single cells around themselves when cell size is artificially increased drastically, revealing the ability of the system to harness diverse molecular mechanisms toward the same anatomical outcome (Fankhauser, 1945). These are but a few of the ubiquitous examples of remodeling toward an invariant end. They reveal the ability of somatic cells to not only pursue specific target morphologies despite perturbations and novel scenarios, but to pursue collective patterning outcomes that are truly enormous with respect to the scale of size and organization of single cells: the length of a limb or the configuration of a face are simply not defined at the level of single cells – the set points of pattern-homeostatic mechanisms implemented by a cellular collective are large, organ-level macrostates.

Also important to the understanding of compound biological individuals is the ability of cells to make decisions as a single coherent unit. For example, in early embryos, regions on the left and right sides of the midline need to express left- or right-specific genes in order to establish invariant laterality of the heart and visceral organs. Experimental interference with a number of physiological mechanisms upstream of asymmetric gene expression is sufficient to randomize this normally invariant pattern, producing duplicated (LL or RR) or reversed (RL) patterns. Remarkably, however, this randomization is made on a *group* level – in each case, the entire domain randomly picks L or R identity, not exhibiting a speckled appearance where each cell adopts a stochastic outcome. The decision is random, but all of the cells in the domain are coordinated to make the same random decision (Levin et al., 2002). This is likewise seen in the conversion of normal frog pigment cells to melanoma – the decision to convert is stochastic across a population of animals, but it is made by all of the melanocytes within a body acting as a single coherent deciding unit (Lobikin et al., 2015). The ability of cells to join into domains that execute group decisions, whether deterministic or stochastic, is an essential component of the evolution of complex forms and will be a central component of a proposed definition of a Self.

The empirical utility of pursuing metaphors based on the parallels between adaptive whole organism behavior and the plasticity of cellular activity during construction and repair of a body is discussed in detail elsewhere (Pezzulo and Levin, 2015). However, for the proposed view of agency described below, and for thinking about its evolutionary origin, it is important to realize that the parallels between goal-directed behaviors and morphogenesis are not only functional but reflect deep conservation of molecular mechanisms. Neural networks control the movement of a body in three-dimensional space; this scheme may be an evolutionary exaptation and speed-optimization of a more ancient, slower role of bioelectrical signaling: the movement of body configuration through anatomical morphospace during embryogenesis, repair, and remodeling (Sullivan et al., 2016; Mathews and Levin, 2018; Mclaughlin and Levin, 2018). This is an expansion of previous proposals of minimal cognition as sensorimotor coordination (Van Duijn et al., 2006), to include cell behavior during morphogenesis as a kind of sensorimotor activity of a patterning Agent.

Developmental bioelectricity is the ubiquitous exchange of slowly changing ion-based voltage signals within and among cells (Funk, 2013; Levin and Martyniuk, 2018). All cells are electrically active, and modern neurons evolved from pre-neural precursors that were already reaping the benefits of ionic signaling for computation. How ancient are these mechanisms – how early was bioelectric coordination exploited by evolution?

Bioelectric dynamics, already used for coordination within bacterial biofilms (Prindle et al., 2015), also enable embryonic tissues to implement robust growth and morphogenesis (Bates, 2015), as well as by unicellular organisms to coordinate behavior (Van Houten, 1979). Recent advances in developmental bioelectricity have shown how endogenous dynamics of resting potential changes modify transcriptional cascades and thereby instruct axial patterning, organ determination, and size control, as well as guiding the behavior of individual cells (Sundelacruz et al., 2009; Levin, 2014; Levin and Martyniuk, 2018). It is thus not surprising that drugs that modify traditional cognitive capacities can be strong teratogens (Hernandez-Diaz and Levin, 2014), while anesthetics reduce and modify regenerative capacity (Buchanan, 1922; Tseng et al., 2010). We have previously argued that the deep evolutionary conservation of ion channel and neurotransmitter mechanisms highlights a fundamental isomorphism between developmental and behavioral processes. Consistent with this, membrane excitability has been suggested to be the ancestral basis for psychology (Grossberg, 1978; Cook et al., 2014; Pezzulo and Levin, 2015). Thus, it is likely that the cognitive capacities of advanced brains lie on a continuum with, and evolve from, much simpler computational processes that are widely conserved at both the functional and mechanism (molecular) levels. The information processing and spatio-temporal integration needed to construct and regenerate complex bodies arises from the capabilities of single cells, which evolution exapted and scaled up as behavioral repertoires of complex nervous systems that underlie familiar examples of Selves. An appreciation of these aspects of developmental biology blurs the distinction between mind and body – a direction already being explored in the field of soft body robotics (Pfeifer et al., 2007a,b; Pfeifer and Gomez, 2009), which expands the possibilities for defining new kinds of Selves. A computational perspective on the task of building specific complex anatomies suggests interesting new ways to think about how an integrated Self can be formed and how its boundaries are maintained or altered during its lifetime and within evolutionary timescales.

## Multicellularity vs. Cancer: The Shifting Boundary of the Self

The communication that enables cells to join into collectives that make decisions about the growth and form of organ-level structures (i.e., what to sculpt and when to stop) can go awry, resulting in cancer (Chernet and Levin, 2013a; Moore et al., 2017). Despite highly diverse molecular and clinical manifestations, one common aspect points to the key: in carcinogenic transformation (Yamasaki et al., 1995; Ruch and Trosko, 2001), cells become isolated from the physiological signals that bind them into unified networks (the essential role of bioelectricity in this process is discussed in the next section). In the absence of global cues, they revert to their unicellular past, when their behaviors were aimed at optimizing the future of just one cell: proliferate as much as possible, and travel to whatever location affords the best local environment for nutrients and expansion (Davies and Lineweaver, 2011; Bussey et al., 2017; Zhou et al., 2018a). This is a breakdown of multicellularity and highlights the fact that the scale of the structure which cells work to maintain can change rapidly – from cooperation toward an entire organ system or body to the level of a single cell.

Normal bodies consist of networks of cells working together toward a unified goal - create and maintain specific large structures. In an important sense, each cell is integrated, *via* physiological signaling, into a coherent swarm intelligence with system-level (anatomical) goals. In cancer, the scope of the coherent Self of a cell reduces from being as large as the boundary of a whole body, to that of just one cell’s surface. The scope of the Self – the structure which a cell works to maintain and support – shrinks drastically in two main ways.

First, it shrinks spatially: being electrically isolated from their neighbors by a shutdown of gap junction synapses, a cell can neither measure distant events nor communicate across anatomical distances with other cells and with the outside world (Kull, 2000; Ay and Lohr, 2015). All of the attention (in terms of measurement and activity toward scaled goals) is focused at the single cell level, while the rest of the body is treated as the “external environment” (and all living things exploit their environment for their own benefit). Thus, the boundary between self and non-self (David Krakauer et al., 2014) can shift: multicellularity enlarges it, while carcinogenesis reduces it, and it is readily seen how this shifts the behavior of cells toward a mode that is not beneficial for the organism level.

Moreover, the size of the Self shrinks temporally: the time horizon of activity shifts from decades (somatic cells execute regenerative and repair processes that maintain a body up to ~100 years) to a much shorter time frame, as cancer cells undertake activity which may kill the host (and themselves) within a mere year. Normal cells pursue goals (organ maintenance) that can be much longer than a cell’s individual lifespan, especially in tissues with rapid cellular turnover – the time scale for such homeostatic activity is one that belongs to the collective, not the cells themselves. Shrinking their Self reduces the ability to work toward temporally distant goals (reduces their temporal horizon of concern) and, together with the inability to communicate as part of a distributed electrical network, makes them literally short-sighted – undertaking activity that will result in death of themselves as well as of the organism (except in the very rare cases of transmissible tumors). This view predicts that interference with (or restoration of) physiological communication among cells should be able to trigger (or suppress) cancer and that the relevant parameter (communication) is spread out over considerable distance and not confined to single cells (e.g., genomic damage). The general fact that actively patterning (embryonic and regenerative) environments can reprogram cancer cells to normal histogenesis has been known for decades (Illmensee and Mintz, 1976; Kasemeier-Kulesa et al., 2008; Oviedo and Beane, 2009). However, the specific prediction of a role of real-time bioelectric communication in cancer has been confirmed *in vivo* in recent experiments: metastatic transformation of normal melanocytes can be achieved in genetically normal tadpoles simply by depolarizing a specific cell population (Lobikin et al., 2012), while, conversely, human oncogenes which normally induce tumors can be prevented from doing so simply by optogenetic or constitutive hyperpolarization (Chernet and Levin, 2013b, 2014; Chernet et al., 2015, 2016).

One important aspect should be stressed, in connection with the classic evolutionary concept of “selfishness” (Werren, 2011; Kourilsky, 2012): on the above-mentioned view, the cells in a metazoan organism body are *not* less selfish than unicellular organisms or cancer cells. They are equally selfish, working only for the benefit of themselves, but the relevant *self* that they defend is bigger in terms of space, time, and complexity. As will be argued below, what defines this Self is the boundary of information being able to pass between the subunits. It should be noted that this change is not binary and can occur at intermediate levels, e.g., teratoma tumors have coherent differentiated tissues (teeth, hair, skin, and muscle) but are not functional embryos, illustrating a level of integrated organization between that of cells and whole organs. It is an essential aspect of Scale-Free Cognition that most biological systems consist of *multiple*, nested selves [not one, as implied by Integrated Information Theory (Tononi, 2008)]. Each inner agent maintains its own local Self only to the extent that it regulates (restricts) information flow from neighbors and thus establishes unique states that do not coincide with those of the collective. Evolutionary and game-like dynamics occur within and between each level of organization, *via* cooperation and competition. While competition between cells in a body is only now beginning to be characterized in molecular detail (Gogna et al., 2015), the remarkable fact that different substructures in an otherwise unified organism do not all have the same goals and can work at cross-purposes was realized already at the very dawn of developmental biology by Roux, who presciently wrote of the *struggle of the parts* in an embryo (Heams, 2012).

Some biologists model cancer cells as individuals, while some see a tumor as its own organ (Egeblad et al., 2010), which can take over other cell types (Gabrilovich, 2017) in the same way that embryonic instructor cells control others in normal development (Vieira and McCusker, 2019). These possibilities could now be distinguished experimentally and analytically using advances in information theory that enable rigorous comparison of the causal power of models at different levels of description (Hoel et al., 2013, 2016; Moore et al., 2017). Identifying the most efficacious level of organization in a given system is critical for selecting targets in biomedical or engineering approaches to understand and control it. Is the best strategy chemotherapy, which seeks to identify and destroy irrevocably broken cells, or could there be normalization strategies that re-connect cells into a collective? The latter is a tantalizing possibility, as it has long been known that embryonic or regenerative environments can reprogram tumors *in situ* (Mintz and Illmensee, 1975; Kulesa et al., 2006). Motivating a system with inputs and experiences at the appropriate level is always easier than attempting to rewire complex systems with emergent dynamics in attempts to micromanage specific global outcomes. Understanding the decision-making, not only the molecular mechanisms, is essential and is perhaps one path toward resolving the unsatisfactory state of cancer medicine (Soto and Sonnenschein, 2013; Sonnenschein and Soto, 2015). Similar approaches are being taken with respect to swarms of insects, robots, and human beings (Couzin, 2009; Deisboeck and Couzin, 2009; Gomes et al., 2013; Rubenstein et al., 2014).

The ability of subunits to enter into a communication network with variable scale of boundaries is already being recognized in swarm dynamics, where the termite nest “superorganism is marked by a kind of extended physiology”, which supports regenerative repair (taking the large termite nest as the “body”), a kind of swarm cancer, and primitive cognition functions such as hypothesis testing carried out by the swarm during the repair process (Beekman and Oldroyd, 2008; Turner, 2016). As will be seen in the next section, this continuum between somatic pattern and cognitive systems reflects a deep scale invariance of cognitive concepts that apply as much to societies of cells as of societies of animals (Seeley, 2009; Turner, 2011). The view of the extent of active information as central to demarcating what exactly constitutes an Individual Self in a given context, especially the focus on a set of level-specific goal states that a system is able to pursue, suggests a formalism for identifying, categorizing, and comparing highly diverse types of Individuals.

## Defining Individuation From a Cognitive Perspective

“By the term “mind,” I mean ideas and purposes.”

–McCulloch, 1951

I propose a definition of an Individual based on its information-processing structure (Barandiaran et al., 2009): the scale and types of goals that a system can pursue defines (determines) the boundaries and content of the putative “agent.” On this view, what defines a coherent, unified Self out of its constituent components and the surrounding environment is the set of parts that operate toward reaching specific goal states. Like a theorem, which has a holistic nature not shared by any of the axioms alone, a functional individual is more than the sum of its parts in the sense that goal-directed capacity emerges only from the integrated activity of all of the components (Weiss, 1967). This flows naturally from the conception of a goal-seeking system: having specified goal state(s), it is immediately clear than there must be an integrated system that can perform all of the parts of the Test-Operate-Test cycle, which may be invisible by inspecting each of the components in micro detail. This definition is orthogonal to other definitions, e.g., evolutionary or genetic ones, but having bigger goals (such as organ morphogenesis instead of single-cell proliferation) de-Darwinises cells and Darwinises groups, shifting the locus of selection and competition (Michod, 2007; Godfrey-Smith, 2009). I propose a working definition for the degree to which a system is a coherent Self, in terms of the goals (in the cybernetic sense) that the system seeks to achieve. Thus, a system’s ability to operate toward specific counterfactuals (future states that are not true right now but can be brought about through specific actions) is central to defining, understanding, and communicating with an arbitrary Individual, which might consist of many levels of organized components.

Complex cognitive systems can have very large and multifaceted goals, and this arises through evolutionary and ontogenetic scale-up and elaboration of primitive goals that arose from constraints of thermodynamics and homeostasis. The initial, most primitive feature of a living system is preferences – the fact that some states of the world are better for its welfare than others. This enables learning from positive and negative reinforcement, which leads to an explosion of computational and behavioral capabilities. Preferences evolve into goals to the extent that a system grows in complexity and causal power and becomes able to act in the world in ways that are likely to move it toward preferred regions of its state space (initially, focused on simple metabolic survival, but ranging all the way to complex human psychological needs and perhaps beyond). It is likely that any life we observe today (which has passed the filter of selection) has preferences and is good at optimizing for them (making this criterion a useful part of the very definition of life), but it is possible that our initial efforts at artificial life may construct some truly primitive, transitional cases that can maintain a degree of coherence in a sheltered laboratory environment without an efficient goal-seeking capacity.

A very simple organism can only have preferences about what is occurring at the current time, in its immediate environment. A more complex organism whose causal structure enables associative learning can pursue or avoid stimuli that are several steps removed in space, time, and causal connection from whatever it is choosing among. This kind of learning enables associations between stimuli that impinge on very different sensors in different parts of the organism and leads to behavioral preferences about stimuli that are in themselves neutral (do not cause damage or provide immediate reward) but are linked to future positive and negative outcomes by past experience (indirect causal connections stretching across body distance and life history).

Others have already made the link between associative learning and the emergence of self (Ginsburg et al., 2019). Very complex selves can have preferences about abstract states that are very far away indeed (such as human beings who are genuinely troubled by the ultimate fate of our star and are actively working toward long-distance space travel and the fate of humanity as a whole). However, the spectrum of Selves is not a simple linear one because of the very wide range of possible natural and artificial agents we do (and will increasingly) encounter. The biological world offers numerous corner cases of swarm individuals, but a good conceptual framework will also include organic artificial life, engineered (computer-based) artificial intelligences, and potential exobiological discoveries. Can highly diverse Selves, with very different material structures be compared with each other in any meaningful way? I suggest that a universal rubric, applicable regardless of the physical implementation, can be defined by focusing on the information processing and goal-directed activity of any given system.

**Figure 2:**
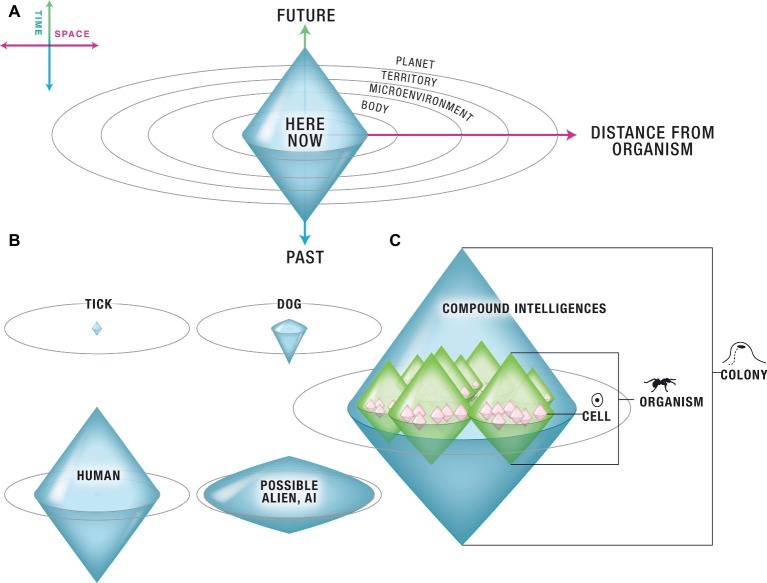
Arbitrary cognitive “Individuals” can be classified according to their computational boundary. **(A)** Each living system has a delimited “area of concern” – a region of space-time, with the organism at its center, within which its cognitive apparatus functions to take measurements and act. The borders of its cognition are schematized on a semi-quantitative state space defined as follows. The vertical axis is time. Values below the individual’s Now are past events, of which it may have a memory extending some duration in the past; values above the Now are future events, which it may be able to predict or anticipate to some distance in the future. The horizontal axis represents three dimensions of space. Each individual, based on its sensory and effector apparatus, and the complexity and organization of information-processing unit layers between them, can measure and attempt to modify conditions within some distance of itself. **(B)** The size and shape of this cognitive boundary defines the sophistication of the agent and determines the scale of its goal directedness. This scheme enables multiple agents, regardless of their composition/structure or origin (evolved, engineered) to be directly plotted on the same space. The shape of boundary defines each agent’s “cognitive light cone” – anything outside this region is mentally inaccessible to that system. Here are illustrated a few representative life forms. Primitive agents such as ticks may only have a very small area within which they can sense signals and operate – immediately next to them, and without much memory or ability to anticipate future events. Dogs have significant memory, but very limited ability to plan for the future and can only really care about events in their local vicinity (it is not possible to get a dog to care about what will happen several miles away, or in 2 weeks). Humans exhibit a great diversity of cognitive boundary shapes but on average have a memory that lasts ~10^2^ years, can anticipate decades into the future, and often plan and act to attempt to modify events on quite distant spatial scales (sometimes planetary or even beyond). A variety of as-yet unknown alien, engineered, and bio-synthetic life forms could occupy every conceivable corner of this option space. **(C)** In this scheme, Individuals can overlap – the same biophysical system can support a number of coexisting, coupled Selves with different cognitive borders. A coordinated swarm of animals, the individual animals themselves, their organs, their cells, and even the metabolic and transcriptional networks inside the cells each have their own cognitive horizon. They cooperate or compete based on specific circumstances and each can be addressed semi-independently because of the differential goals they pursue (and thus, the different positive and negative reinforcements that can be brought to bear to modify events at a given level). All panels courtesy of Jeremy Guay of Peregrine Creative.

The cognitive boundary of an Individual [a “Center of Concern” (Murase and Asakura, 2003)] is the most distant (in time and space) set of events that this system can measure and attempts to regulate in its goal-directed activity. It is a surface indicating what things this system can possibly care about (conversely, it defines preferences as the spatio-temporal domain of states that serve as inputs and outputs to the system). In advanced agents, it is also the boundary of the self-model. A range of such systems is illustrated in Figure 2: using one axis for time and one axis to represent three dimensions of space enables a semi-quantitative representation within which individuals of very variable cognitive capacity can be plotted in the same virtual space. Recent work in artificial life has already begun to characterize the cognitive domains of very diverse kinds of complex systems (Beer, 2014).

The edges of a given Agent’s goal space define a sort of “computational light cone” – the boundaries beyond which its cognitive system cannot operate. For example, a tick has a relatively small cognitive boundary, having very little memory or predictive power in the temporal direction, and sensing/acting very locally. A dog has much more temporal memory, some forward prediction ability, and a degree of spatial concern. However, it is likely impossible for a dog’s cognitive apparatus to operate with notions about what is going to happen next month or in the adjacent town. Human minds can operate over goals of vastly greater spatial and temporal scales, and one can readily imagine artificial (organic or software-based) Selves with properties that define every possible shape in this space (and perhaps change their boundaries over evolutionary and individual timescales). As will be seen in the next section, expanding the horizon is what enables information (in the Shannon sense) to acquire meaning, because data become causally linked to distant and past experiences, and acquires implications for future expectations. The formalism also suggests semi-quantitative definitions of maximum cognitive rate (the speed at which information propagates across an agent’s body); this and other similarities to the space-time diagrams of Relativity remain to be explored.

Clearly, much more work is needed to fully flesh out this rubric in a way that makes it immediately applicable in ethology, AI, and artificial life. One implication of this view is that there is not necessarily one unique primary level of organization; rather, Individuals existing at different levels in a given system can be putatively revealed by experiment and analysis that identifies allostatic set points - the goals of systems defined at different levels of organization and the spatio-temporal boundaries of measurements and actions taken by such mechanisms at each level. It is of course non-trivial empirical work to ascertain goals, especially for novel or alien ones. Moreover, systems and subsystems will have different, overlapping, and not always aligned goals. Thus, another key implication is that systems house multiple levels of coexisting coherent Selves.

Presumably, the volume of possible cognitive light cones keeps expanding with evolutionary time (although some lineages can contract it, permanently or during specific ontogenetic life stages). Over evolutionary scales, there have been “inflationary leaps” – innovations in body structure which drastically increase the cognitive boundary of viable selves. The formalism places no upper bound on the size of the cognitive light cone. Thus, while we understand “diminished capacity” for human beings in a legal and psychological setting, it is important to also consider “increased capacity”: what would a being with a much larger cognitive boundary than a typical *Homo sapiens* be like? It is interesting to consider whether this is the sort of outcome envisaged in classical traditions that posited evolution of human consciousness to a state of “Buddha-hood” – a cognitive boundary so large that such an individual would be capable of, for example, being directly concerned about the individual welfare of a myriad of beings. It is likely that efforts in artificial life, biomedical enhancement, or engineered AI will eventually give rise to much larger Selves, since it is unlikely that today’s human cognitive sphere is the maximum possible one.

Next, we consider a possible sequence of evolutionary scenario for the expansion of cognitive boundaries.

## The Agent’s Evolutionary Back-Story: Scaling of Information by Bioelectricity

What evolutionary pressures lead cognitive boundaries to expand, resulting in the variety of agents observed in the biosphere? A sequence of phases can be hypothesized (setting aside the abiotic origin of chemical systems with feedback). I propose that the “atom” of this cognitive hierarchy is homeostasis (Bernard, 1865; Cannon, 1932; Wiener, 1961; Man and Damasio, 2019). The ancient origin of living forms is predicated on the ability of some simple systems to achieve coherence and distinction from the external environment, against perturbations, maintaining spatial and metabolic integrity by ensuring certain parameters stay in specific ranges (Maturana and Varela, 1980; Luisi, 2014). This homeostatic persistence is the origin of cognitive goals – the setpoints of subcellular biochemical circuits that react to perturbations in a way that maintains parameter range are the first, tiny examples of integrated goal-seeking systems. Homeostatic setpoints (actually, ensembles and not single points) are encoded by biophysical properties that guide activity toward implementing those states. The ability to operate toward a region in state space may be the primitive origin of complex cognitive systems that can entertain counterfactuals (remember or anticipate events that are not occurring right now).

Minimization of anti-homeostatic stress is a powerful driver, already suggested to be an important factor in evolutionary innovation (Erkenbrack et al., 2018; Miller et al., 2018; Wagner et al., 2019). A second step to the simplest homeostatic loop is the inclusion of a richer set of “hidden layers” (in the neural network sense) - additional biochemical nodes between the sensors and effectors of a given system that enable a degree of memory. By introducing a delay between inputs and outputs (Flack, 2012, 2017) and by including additional feedback loops that can maintain state after transient stimuli, a much more powerful homeostatic circuit is created, which uses memory of past events to anticipate, not merely react to, environmental challenges. The molecular basis of such anticipatory circuits, and biochemical machinery that responds to different frequencies in signals, has been described in single cells, such as ERK signaling dynamics and metabolic networks (Toettcher et al., 2013; Bugaj et al., 2018; Katz, 2018). Chemotaxis, such as seen in bacteria, is an early example of this because it exploits time delays in environmental sensing as a kind of memory to enable prediction/anticipation that enables it to optimize travel up nutrient gradients (Vladimirov and Sourjik, 2009; Scherber et al., 2012). The earliest forms of such memory are direct and not representational, e.g., slime molds learn to cross salt bridges to get rewards by storing the salt itself in their bodies as the engram of the memory used to guide future decisions (Vogel and Dussutour, 2016; Boussard et al., 2019).

More advanced creatures possess richer networks that allow complex forms of learning in which memories and goal states are implemented by settings (configurations) of internal mechanism several biochemical steps removed from the actual state in question (perhaps the origin of symbolic representation in more complex minds). Reactive homeostasis evolves into predictive allostasis (Schulkin and Sterling, 2019), under the pressure to predict signals from environment and other elements of the biosphere (Seoane and Sole, 2018). Importantly, memory can serve as the beginning of modularity because learning essentially groups diverse stimuli into compressed representations: complex states of affairs become remembered as compact biophysical engrams – this is the essence of the kind of modularity when a simple biophysical event kicks off the formation of a complex morphogenetic cascade such as building a “hand” in embryogenesis. In associative learning, some complex state gets functionally “hooked” to a trigger stimulus; this stimulus may be much simpler (in terms of information content) than the state it represents or the activity that it will kick-start. In somatic control networks, this is implemented as developmental modules – complex downstream morphogenetic activities that are initiated by a single driver. It has often been noted that modularity confers benefits for evolvability (Schlosser and Wagner, 2004; Wagner et al., 2007), but it is not always appreciated that like many other key aspects described above, it has an origin continuous with (and is driven by) control and proto-cognitive capabilities of the simplest living agents. The recent discovery that very simple bioelectric states can trigger complex organ formation, such as induction of complete eyes in various parts of the animal (Pai et al., 2012), are consistent with a primary role of bioelectric signaling in both learning and morphogenetic control mechanisms (Levin, 2012).

The sensory machinery, implementing inputs to the cognitive agent, is extremely ancient. Complex animals’ sense of touch, taste, temperature, hearing, etc. are mediated by the exact same ion channel mechanisms that were already discovered by bacteria and used to make sense of their world (Tasneem et al., 2005; Blackwell, 2006; Liebeskind et al., 2012; Nilius and Honore, 2012). A rich sensory input layer, consisting of receptors and channels on a cell’s membrane enables the dynamics of Active Inference to operate (Friston et al., 2012; Pezzulo et al., 2015), and is itself highly regulated by cellular transcription and translational machinery in accordance with the main tenet of Perceptual Control Theory: behavior is the control of perception (Powers, 1973). Systems minimize surprise and optimize variational free energy by tuning their internal states in a way that optimally predicts future stimuli (Friston and Ao, 2012; Friston, 2013; Badcock et al., 2019). This is as true for nervous systems as it is for individual cells during metazoan development (Friston et al., 2015; Ramstead et al., 2019). This kind of predictive coding is data compression – a mechanism that works to represent a long history of environmental inputs into a compact representation *via* tuning of internal states. This basic learning and inference capability is the primal origin of “understanding” and intelligence in advanced brains, which can be defined as the process of inferring patterns from raw data and making “maps” of regularities in observations that occupies every Self from the simplest of organisms to the scientist working on extracting deep theory from data (Dingle et al., 2018).

The advent of multicellularity has been proposed to result from the drive to minimize surprise: a cell need only to surround itself with its progeny, in order to ensure a much more predictable milieu (the least surprising object in the world is a copy of yourself) (Fields and Levin, 2019). If these “front line infantry” are kept in place and suppressed from proliferating (and differentiated), one immediately gets the kind of arrangement seen across biology, from stem cell/soma systems to queen/worker dynamics in insect colonies. The surrounding body becomes an informational shield (Markov blanket) for the stem cell (Friston, 2013); adding epithelial and mesenchymal cell layers enables a kind of neural-like network in which each layer provides an increased level of abstraction from environmental stimuli, enabling deep tissues to perceive not simply raw signals but highly processed, dimensionally-reduced, compressed data that form a kind of primitive “knowledge” about the outside world’s patterns.

**Figure 3:**
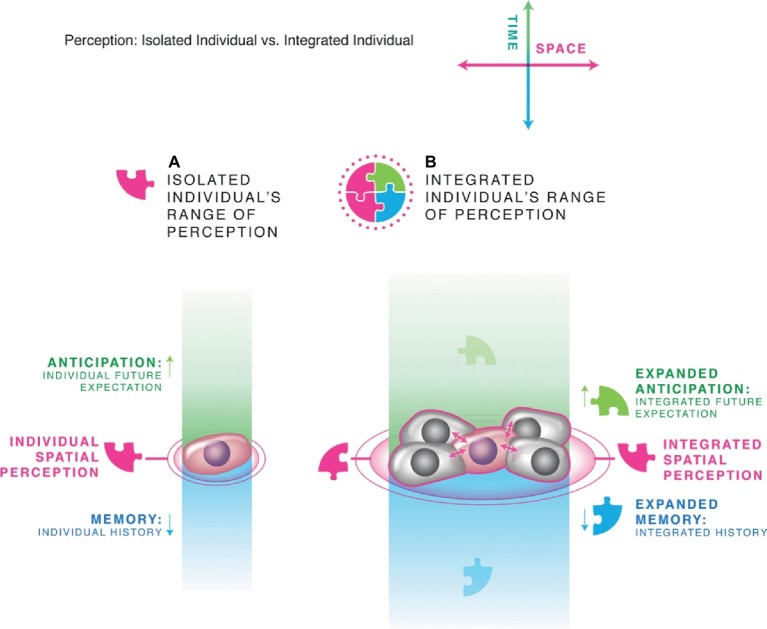
Scale-up of cognition. This schematization uses the example of single cells vs. a gap-junctionally coupled tissue, but the same basic system is applicable to many scales and many types of agents, both engineered and evolved. **(A)** A single small agent, like a cell, has limited spatial and temporal perception. It is able to infer, store, and operate with respect to a small subset of the patterns existing in its environment (symbolized by the single pink puzzle piece). **(B)** Joining into communicating networks allows each cell to have (self-regulated and temporally delayed) access to the information obtained by neighboring cells, as well as to form layered architectures with progressive layers abstracting patterns from raw data. In this way, the subunits not only expand their spatial range of perception but also can improve memory and a degree of predictive ability. The resulting larger Individual is formed with a cognitive world that is unified by the cells’ sharing of information across time and space. All panels courtesy of Jeremy Guay of Peregrine Creative Inc.

**Figure 4:**
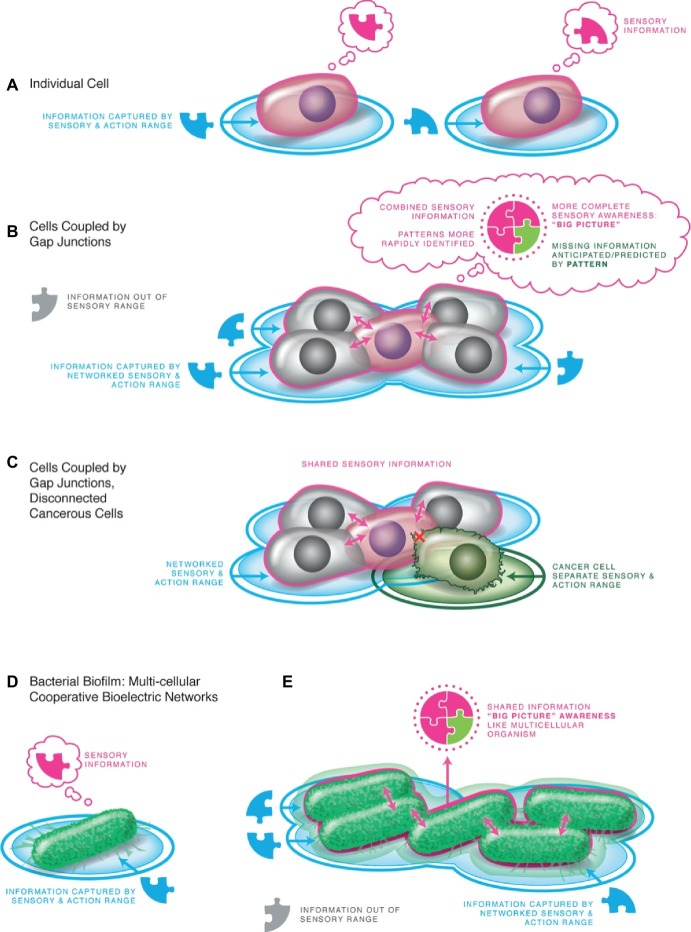
Multicellularity: integrated Individuals arise from sharing bioelectrical information. **(A)** An individual cell perceives its local environment, and distinct cells perceive different local conditions. **(B)** When joined by gap junctions (electrical synapses), all of the cells have access to the same information, able to perceive states from the edge of the collective. This results in the ability of the system to react to and manipulate the large-scale environment in its homeostatic cycle. The ability to pursue spatio-temporally larger goals instantiates a bigger Individual with a different cognitive structure than any of the subunits. The sensory and set point conditions are scaled to the whole body, enabling maintenance of large anatomical features such as morphology. **(C)** The physiological coupling process can exhibit local breakdowns by loss of gap-junctional communication (which can be induced by oncogene activity) or by introducing physical barriers (which are known to induce cancer without any prior genetic damage). Individual cells thus disengage from the larger Individual, shrinking their cognitive horizon and reverting to their unicellular past resulting in over-proliferation and metastasis as the cell pursues its cell-level goals of individual survival. **(D)** Bacteria already had ion channels such as those used by metazoan cells, exploiting them and chemical receptors to sense their local environment in the same way that complex animals and plants use them as sensors. **(E)** Bacterial biofilms are proto-bodies, already using bioelectric signaling to enable time-sharing of nutrients across the collective – an early form of tissue-level integrated goals (Prindle et al., 2015; Humphries et al., 2017; Lee et al., 2017; Liu et al., 2017). All panels courtesy of Jeremy Guay of Peregrine Creative Inc.

This transition from single cell state to multicellular tissue is a crucial step in the expansion of the cognitive boundary and the creation of more complex agents with larger goals (Figures 3, 4). By receiving information from neighbors, a cell in the center of a tissue can get (filtered) data about events occurring at a considerable distance. Because of the finite spread of signals through tissue and the predictive capacities of networks, this expands the cognitive horizon of a cell in both space and time. Crucially, by organizing into a (partial) electrochemical and informational pool or syncytium, all of the cells are able to measure and detect events occurring within *the same boundary*, creating a larger individual that emerges from the collective. The new Individual is an integrated whole because its subunits are no longer exclusively have their own local, distinct microenvironments (and thus internal models of the world) but are constrained to share a bigger, common reality due to the spread of information and influence among them. Such collective individuals can have a higher problem-solving capacity than their members, because they can support a layered architecture with experience-dependent communication channels (synapses, broadly defined), emergence of “virtual governors” with beneficial properties with respect to control capacity (Dewan, 1976), and more complex state space with more attractors and thus can compute meta-system properties not accessible to the single agents (Hofstadter, 1979; Crutchfield et al., 1998; Cenek, 2011).

It is proposed that morphological complexity and multicellularity are driven by greedy infotaxis (Vergassola et al., 2007) – collecting as much information as possible, from as far away (in both space and time) as possible, to minimize surprise and optimize prediction. The more a cell is connected to other cells in networks, the more processing capacity and the bigger the horizon of what the compound individual can potentially sense, remember, and store (Crutchfield et al., 1998). Merging into communicating networks also enables more noise tolerance and robustness – the field of artificial neural networks exploits the fact that some kinds of networks facilitate extracting patterns from data and can ignore irrelevant details while picking up on salient patterns. Inevitably, the scope of the Self expands with this integrated, growing cognitive domain. What molecular mechanisms might underlie this in cellular systems?

A fundamental facilitating step in this process is provided by developmental bioelectricity: the exchange of ionic signals within and among cells to propagate instructive influence at multiple scales of organization (Levin, 2012). Electric circuits are very convenient for processing information (a fact that has not escaped the attention of computer engineers and evolutionary mechanisms of neural systems), because they facilitate integration of information over spatial domains, and form feedback loops (such as voltage-gated ion channels) that readily implement memories and homeostats (Pietak and Levin, 2017; Cervera et al., 2018). This proposal, of ancient bioelectrical systems of coordination at the dawn of multicellularity, has now been confirmed by elegant studies of brain-like integration of spatial information by ion flows in the proto-bodies known as bacterial biofilms, which show how group-scale goals can emerge in simple physiological systems (e.g., nutrient sharing in the bacterial collective) (Prindle et al., 2015; Humphries et al., 2017; Lee et al., 2017). In more advanced cell types, this same scheme is implemented not by extracellular ionic waves but by intracellular electrical synapses known as gap junctions – highly regulatable valves that allow cells to selectively share bioelectric state with neighbors and implement activity-dependent plasticity (memory) (Palacios-Prado and Bukauskas, 2009; Zeng et al., 2009; Mathews and Levin, 2017).

Individual cells will all measure local conditions at their cell surface. When coupled into networks *via* gap junctions however, the whole collective can measure and act upon the same sensory data – the Umwelt expands to create a larger individual in which the comprising cells share a unifying picture of the world (Figure 4). It is thus no coincidence that most anesthetics used to remove cognition and sensory experience, whether in plants or animals, are gap junctional (bioelectric) uncouplers (Mantz et al., 1993; Wentlandt et al., 2006; Liu et al., 2012; Gremiaux et al., 2014; Baluska et al., 2016). Thus, physiological connectivity is the binding mechanism responsible for the appearance of larger unified Selves. The coordination of cells toward a single goal (body patterning) is now known to be in part controlled by the activity of bioelectric networks mediated by gap junctions (Levin et al., 2017; Mathews and Levin, 2018; Mclaughlin and Levin, 2018; Pietak and Levin, 2018). Consistent with the isomorphism between patterning control and behavioral control, the same scale-up occurs in morphogenesis: subcellular chirality that determines morphology of single-cell organisms is amplified during early development by a system of gap junctions and ion channel-driven bioelectrical cues into animal-wide axial patterning (the appearance of a new anatomical level – “whole body symmetry”) (Levin, 2003, 2006).

The developmental control bioelectric network shares mechanistic evolutionary history with the nervous system, and the conservation extends not only to mechanisms of targeting connections [such as neural-like mechanisms deployed in plant pollen tubes (Palanivelu and Preuss, 2000)] but to the ubiquitous use of neurotransmitter molecules downstream of bioelectric driving forces [e.g., GABA and the serotonin-like Auxin used in many plants (Ramesh et al., 2015)]. An intermediate form between somatic bioelectric networks and neural networks sheds light on how local gap junctional connections in tissues were refined into more targeted connections on the scale of several cell diameters: tunneling nanotubes. This axon-like structure present in many cell types is an intermediate cellular appendage that has a gap junction at its end, allowing limited directed connections between cells in a tissue, and mechanistically presaging neural axons and their electrical synapses (Wang et al., 2010; Sherer, 2013; Ariazi et al., 2017). The general bioelectric system was speed-optimized during the development of nervous systems for behavior (Van Duijn et al., 2006; Keijzer et al., 2013; Jekely et al., 2015), but uses fundamentally the same set of mechanisms to optimize the input-output relation within and between the internal milieu mechanisms and the outside world (Molnar-Szakacs and Uddin, 2013; Fields et al., 2019). Increasing the correlation length of such control could have driven the development of targeted, long cellular structures (axons): to move the whole animal, not just individual cells, the electrical information needs to be scaled considerably, and nervous systems are an ideal extension of ancient, pre-neural bioelectric signaling paths to enable further scaling of the cognitive boundary of cells into organs, organisms, swarms, and societies. A prediction of this proposed scaling is that swarm organisms should fall prey to the same kinds of cognitive illusions and specific failures of rationality as do vertebrate brains, which has indeed been observed in ants and slime molds (Sakiyama and Gunji, 2013, 2016; Tani et al., 2014; Beekman and Latty, 2015). Another consequence is that the cybernetics of associative learning in networks is agnostic as to the spatio-temporal scale and physical implementation, being widespread from molecular networks and inorganic physics to whole evolving populations (Cragg and Temperley, 1955; Mcgregor et al., 2012; Power et al., 2015; Watson and Szathmary, 2016; Kouvaris et al., 2017; Lopez Garcia De Lomana et al., 2017).

## Conclusion and Future Outlook

“The self is not something ready-made, but something in continuous formation through choice of action.”

–John Dewey

A number of ideas from cognitive neuroscience, information theory, computer science, cybernetics, and engineering converge on key questions in biology with respect to the multi-scale interface between body structure and mind. The scheme described above seeks to (1) define Individuals and Selves in a way that facilitates taxonomy, comparison, and communication with evolved, created, biological, artificial, and exo-biological agents, and (2) propose a plausible naturalistic framework for the evolutionary scale-up of cognition from earliest origins of life, hypothesizing about the forces that drove it and the major transitions along the continuum. The goal of this research program is to show how complex agency and goal-directedness evolves naturally from ancient mechanisms. The evolutionary pressure to survive in a challenging world leads (in order) from simple homeostasis to infotaxis, memory, anticipation, spatio-temporal scale-up of measurement and prediction, and large-scale global goals (system-level agency). The implications of these hypotheses extend beyond philosophy and evolutionary biology. Practical strategies for regenerative medicine (control of cell collectives *in vivo* toward macrostates such as “healthy structure and function”), exobiology, and robotics/AI are impacted by our view of what defines a coherent Self.

BOX **Summary of key ideas of Scale-Free Cognition**

1. A unified, integrated cognitive Self or Individual can be defined with respect to the integrated ability to pursue specific goals *via* a homeostatic process that resists perturbations. Goals also define positive and negative reinforcement for that agent, thus enabling communication with/training of highly diverse intelligences. The ability to pursue goals is a major ratchet for evolution because it smooths the selection landscape: the potentially destructive effects of individual mutations are often made up for by the regulative ability of other mechanisms to accomplish specific outcomes despite changes of circumstance (e.g., cells providing blood vessels and tendons to make a coherent finger when a new hand bone is induced).
2. An Agent’s cognitive world can be quantified and characterized, enabling comparison with others (regardless of their material implementation), by estimating the spatio-temporal boundaries of its area of concern: the volume in space and time over which the agent is able to take measurements, exert influence, and functionally link disparate events (learning, association).
3. The borders of the temporal and spatial events of which a given system is capable of measuring and acting map out a “cognitive light cone” – a boundary in the informational space of a mind. These borders can grow or shrink, on evolutionary or ontogenic time scales, as the organization of an agent changes. The key is a balance of selective information sharing, *via* “synapses” – structures of arbitrary physical construction which share the feature that they can regulate the passage of signals based on the state of other such elements. Too little sharing results in a failure to bind subunits into a new Self. Too much sharing results in a homogenous soup with insufficient differentiation of modules and abstraction of information across distinct layers.
4. Cancer is a (reversible) shrinking of the computational boundary of a biosystem: by isolating itself from the surrounding tissue’s physiological signals, a cell’s cognitive boundary shrinks to the small size it had before multicellularity. Cancer cells are not more selfish than somatic metazoan cells – they are equally selfish but their Self is now scaled down to a single cell (which will reproduce and migrate as much as it can), whereas the normal physiological binding in healthy tissues binds each cell to a common goal represented by the large network – the construction and repair of a specific large anatomy. These ideas connect naturally to gene-level views of selfishness (Dawkins, 1989); if it is useful to think of genes as selfish agents, it is doubly plausible to view cells and tissues as such, since the latter have much more capacity for activity and computation. Future work will develop, in the contexts of ontogeny and evolution, how the optimization of specific states of affairs (pursuit of goals – selfish or otherwise) occurs simultaneously at many scales of biological organization.
5. Agents scaled up by evolving from basic homeostatic loops, driven by active inference (surprise minimization) *via* addition of delays (memory), anticipation (inference), and networks (spatially-distributed processing that enables learning and progressive abstraction/generalization from data). Gathering into larger collectives with optimal informational structure (Klein and Hoel, 2019) improves the computational (predictive) capabilities and gives rise to functional relationships (memories, encoded goal states, test-operate-test-exit loops) that exist over and above any individual member (Miller et al., 1960). These levels coexist, enabling numerous coherent Selves of different scales to be implemented by any collection of living matter.
6. Infotaxis (the drive for better actionable intelligence about the regularities/patterns in the world, and in the agent’s own mechanisms) encourages cells to connect in groups *via* signaling. On the cellular level, this is implemented by diffusion (bacterial biofilm proto-bodies) or direct connections *via* gap junctions or neurons.
7. Collecting into a syncytium enables all of the cells to share the same data and access the same memories (illustrated, e.g., by the ability of trained slime molds to pass on information to naïve hosts by fusion [Vogel and Dussutour, 2016)], This shared information structure extends to the edges of the large collective, which binds small, individual competent sub-agents into a larger unified Self. These principles likely apply beyond cells in organs, to swarms of whole organisms such as bees and termites (Seeley, 2009; Turner, 2011), as do the dynamics of breakdowns in coordination which share important similarities for example between cancer and social insect colonies (Amdam and Seehuus, 2006).
8. The hypothesis of scale-free cognition does not rely on cooperation *per se* – it builds up apparent cooperation from selfish agents minimizing their stress (surprise) and competing for information. Greed, at the single-cell level, for information (infotaxis) drives cooperativity, as each unit expands its measurement boundary (communication with neighbors) and thus inevitably becomes part of a bigger self with bigger set points serving as homeostatic attractors. It only looks like cooperation from a perspective of a higher level, because the higher level of organization shows an integrated Self which appears, necessarily, cooperative.
9. There is a fundamental symmetry between anatomical control mechanisms and cognitive mechanisms. Co-evolution and exaptation drove the mutual enlargement of mechanisms that control patterning and behavioral goals. The same dynamics operate in unicellular systems, multicellular systems, and colonial/swarm organisms and most concepts from memory to cancer are found at every level of organization in biology, from memory in transcriptional networks to regeneration of termite nest structures.
10. Neurons utilize bioelectric computational strategies that were discovered and exploited by evolution as far back as bacteria. There are no sharp distinctions between neural networks and non-neural somatic bioelectrical networks (although they function on different time scales). The functional isomorphism between patterning and cognitive processes is also reflected in the ancient molecular conservation of mechanisms: ion channels and neurotransmitter molecules are ubiquitous across the tree of life. Bioelectric integration helped evolve control strategies and cognitive content across the continuum from chemical networks to human minds – it illustrates an important mechanism of early evolution. But clearly, numerous aspects of physics (from stress forces to diffusing chemicals/pheromones) are exploited by evolution, or could be exploited by engineers, across the wide range of possible systems.
11. There is a deep functional scale-invariance between the decision-making of cells in building body structures, the workings of an insect colony, and the integrated behavior of a human “Person”: these are the cybernetic processes of learning and parameter optimization implemented by large numbers of units pursuing infotaxis and homeostatic goals at whatever scale the sensory channels permit.
12. A conceptual unification is proposed as Scale-Free Cognition: one major control knob is the boundaries between self and world. These boundaries are malleable, and can shift at different time scales, to sizes limited by what the underlying hardware supports. This parameter determines the scope of the self and implements the continuum leading smoothly from cell- > body- > swarm. Signaling between animals in an ecosystem (Pais-Vieira et al., 2013; Kingsbury et al., 2019; Zhang and Yartsev, 2019) is not fundamentally different than signaling within the brain – both are examples of information propagating through a network of locally-competent micro-agents. Others have pointed out the parallels between the dynamics of cancer and ecosystem-level degradation (Degregori and Eldredge, 2019). Thus, multiple levels of approach to living systems are *a priori* equally valid, with no unique privilege for the lowest (molecular) levels at which everything looks like a mechanism. In any given example of biology or artificial life, the most appropriate level of analysis or description is to be determined empirically: it is the one that facilitates prediction and control with the least effort (by the experimenter or by the system itself), and gives rise to unified understanding that drives the most novel, robust research programs at the bench.

## Discussion

This view is consistent with Dennett’s stack of homunculi (Dennett, 1991). Here, the bottom homunculus is a basic homeostatic loop, which is required for even the simplest life form to persist against entropy (Varela et al., 1974; Maturana and Varela, 1980). Each succeeding level of that same homunculus has a tiny bit more reach in physical space (how far it measures and how far its signals propagate) and in time (how long it remembers past signals and how far it can anticipate) - the homunculus is not entirely blind but has a horizon within which it can perceive. The bottom homunculus measures very simple, immediate things (chemical concentrations on the surface of a single cell), and has a feedback loop that keeps that one variable within a life-compatible range. The others see a little more and manage slightly more complex allostasis. This naturally scales into “goal-directed systems” and enables massive improvements in evolvability because these components make random moves in genotype space into acceptable (and sometimes brilliant) ones in phenotypic space. The functional modularity enabled by learning and goal-directed feedback loops radically modify the evolutionary search space landscape, because they can improve the phenotypic quality of mutations. Mutations that by themselves would have been useless or harmful to the organism (because they usually adjust one feature without directly implementing the many others needed for functional advantage), can be made workable if the various parts are flexible and alter their behavior to reach the same attractors despite shifting surroundings.

Developmental and regenerative biology are full of these examples, like the rearranged tadpole faces whose organs then move around to make a normal frog face (reviewed in (Lobo et al., 2014; Pezzulo and Levin, 2015, 2016)). Likewise, manipulations that cause an ectopic bone to form in the embryonic hand do not simply result in a single, out of place bone - the nearby muscle cells, blood vessels, and tendons make up for this novel circumstance and build an extra, workable finger. This helps explain Darwin’s original puzzle of evolvability because the body is not a static canvas or merely a set of features emerging from micro-level rules. Morphogenetic goal-directedness is a major evolutionary ratchet, smoothing the anatomical fitness landscape by hiding the otherwise potentially disruptive effects of random mutations. This is a point that has been made in the context of gene-regulatory circuits, whose self-organizing capacity takes a lot of the heavy lifting off of the evolutionary process (Kauffman, 1993).

When the subunits exhibit their own level of competent, local intelligence, many mutations do not need to occur before a feature is useful. One mutation can change how something operates, but the other parts may be able to continue to pursue their same anatomical goal even though conditions have changed. This somatic plasticity is ubiquitous in biology because complex agents inevitably consist of micro-agents that were selected on the basis of proto-cognitive competency. This view of modularity from the perspective of basal cognition complements traditional genetic accounts. This is likely also the source of cognitive plasticity. Tadpoles engineered to develop with eyes on their tails instead of in their usual spot can see quite well, despite the fact that the eyes connect to the spinal cord, not the brain (Blackiston and Levin, 2013; Blackiston et al., 2017). Brains, like other developing organs, are not hardwired but are able to ascertain the structure of the body and adjust their functional programs accordingly – a strategy that is already being pursued in robotics, including the use of electrophysiological principles (Bongard et al., 2006; Cheney et al., 2014). This self-discovery phase is an important area for future research (Ramstead et al., 2019).

The way regulatory pathways are organized (primitive intelligence of the parts, exploiting attractors in state space and modularity to attain goals at various levels despite changing conditions) makes complex traits much more easily evolvable. Organs and tissues have local goals (in the dynamical systems and control theory sense of ‘goals’) and their ability to reach these goals despite damage and changing environmental conditions (something for which evolution selected from day one) is what enables random mutations to power evolution in a rapid timeframe. From the perspective of the cells, a mutation is just another attack of a hostile environment - they need to keep going despite this perturbation, and they adjust their activity as they would to any external stressor. On this view, the organization of cybernetic control structures at different levels originating from simple homeostatic survival advantages, are the source of agency at multiple scales (Arnellos and Moreno, 2015).

This view has important implications for treatment of injury and disease (Levin, 2011). Most of the focus of biomedicine today is on the cellular hardware, seeking molecular- or cell-level interventions. The problem is that even with full genomic information and stem cell derivatives, directly creating an organ such as a human hand directly from individual cells is likely to be beyond our capabilities for a long time. Moreover, the complex, emergent nature of morphogenesis establishes an important inverse problem: how to manipulate low-level rules to achieve system level outcomes such as repair or regeneration of organs damaged by traumatic injury or disease? I have previously suggested that a better strategy than cellular re-wiring at the genetic level may be to edit the pattern-homeostatic set point and let wild-type cells build to the new spec. For example, the ability to re-write target morphology in planarian regeneration was recently shown, by altering the bioelectric state in flatworms, without genomic editing, giving rise to permanently double-headed worms (Durant et al., 2017; Levin et al., 2018). Similarly, a brief stimulus (24-h exposure to chemical cocktail) kick-starts a limb regeneration module that drives growth for 11 months with no further intervention (Herrera-Rincon et al., 2018). This ability to initiate very complex, long-lasting morphogenetic cascades *via* a simple trigger demonstrates the utility of understanding the modular control structure of large-scale patterning systems so that the most efficient (least effort, simplest) intervention can be deployed, at the right level of organization, to induce a desired system-level outcome.

What is the difference between a mechanism and decision-making (choice)? This has been widely discussed in the philosophy literature (Barandiaran and Moreno, 2006; Haig and Dennett, 2017) and is now becoming an important issue for bench biologists working in basal cognition of somatic cells and synthetic biology (Perkins and Swain, 2009; Balazsi et al., 2011; Reid et al., 2013, 2016; Mitchell and Lim, 2016; Paul et al., 2016; Vesty et al., 2016; Bugaj et al., 2017) as well as for workers in artificial life (Juel et al., 2019). Without attempting to deal with this profound question in full, it can be mentioned that the perspective taken herein suggests that several factors contribute to a smooth transition between biochemical mechanism and agency: integration of remote events into the causal chain (spatial distance, and temporal distance – memory/anticipation), and stochasticity (distance in terms of predictive capability). The greater the area of the cognitive light cone that comprises the events that cause a particular outcome, the more likely that we will gain explanatory and control power by treating it as a choice and not a push. If an outcome depends on things that happen far away, and in the past (memory) or future (planning), then it is likely to be empirically useful to consider that process a choice. This heuristic has the attractive natural corollary that the bigger the cognitive horizon (scope of self) of an agent, the more apparent freedom that agent has (when causes are very far away, in space and time, from a given action is when folk psychology usually labels an event as a free choice).

Bioelectric signaling is an ancient aspect of physics, which evolution exploited for its computational capacities. Neural Hodgkin-Huxley equations can be derived from first principles of information theory (Gatenby and Frieden, 2017), but the use of bioelectrics long predates nervous systems. Why is bioelectricity, implemented by ion channel and gap junction protein hardware, so well-suited for implementing the flexible software of life? Voltage-gated ion channels and gap junctions are voltage-gated current conductors – basically transistors, from which as we know all manner of advanced computing devices can be constructed. Such channels readily enable loops which either amplify small signals (positive feedback) or implement robustness (negative feedback), because they create voltage changes that then control their own activity (open or close the channel), in a physiological cycle that occurs at the post-translational level (not requiring any gene-regulatory mechanisms). Also, they very naturally link up into networks that enable spatial integration of signaling. Thus, any electrical circuit that enables feedback loops and memory is sufficient for very primitive software behavior in the following sense. It can stably occupy more than one state, and shifting it from one state to the other does not require changing out the hardware (altering protein profiles by transcriptional changes). What makes electric circuits, whether ion channel-mediated or electronic, good for software is that they easily implement dynamical systems that have multiple attractors and can be made to occupy one physiological state or another by transient inputs (signals), not requiring changing the hardware (i.e., mutating the DNA encoding that hardware). This is also true to some extent of physical forces and cytoskeletal controls, which can enable extremely rich morphogenetic and behavioral processing in unicellular organisms (Faure-Fremiet, 1953; Ford, 2017) and can scale up to tissue-level controls (Roper, 2013).

Some agents can transcend one system and re-set its goals from a vantage-point of a meta-system (Hofstadter, 1979). While many animals are capable of a wide repertoire of diverse goal-seeking activity, human minds appear to be (for now) in a unique position. A fish can successfully meet the most basic goal of “survival” because it only sees a short time into the future, and surviving for that short time is usually achievable. However, humans (who are prone to reflection on these issues) can infer that “survival” is not actually an achievable goal on timescales that we can think about; uniquely among goal-pursuing systems, we alone know that this most basic of all life goals is doomed to failure. We are the only known agents who *must* change their goals, because our cognitive horizon is, along the temporal axis, greater than our lifespan and we can envision goals that extend beyond our own life. Therefore, we as cognitive agents are perhaps uniquely motivated to set other goals ahead of survival (Frankl and Vance, 2007). This potentially includes pursuit of the most radical goal of all, to cease functioning as a unified goal-seeking system entirely. “To be, or not to be?” – when did this question first get asked, phylogenetically? A cabbage cannot commit suicide. A human can; could a non-human primate? As with other kinds of behavioral capacities (Bronfman et al., 2016), this capability likely represented an important phylogenetic cognitive transition. Individual cells have this capacity [even unicellular organisms (Gordeeva et al., 2004)]; it is unclear if metazoan organs do. The synthetic biologists may want to ask, how would we create an organism capable of such a meta-goal?

## Predictions and Research Program

Needless to say, many details of this set of ideas remain to be worked out. However, the above-described perspective makes a number of specific predictions which suggest experimental approaches to test and exploit this perspective (Levin, 2011). Future empirical work will reveal whether Scale Free Cognition is a useful way to organize what we know about the mechanisms and algorithms of life and life-as-it-could be (Langton, 1995; Walker and Davies, 2013). Directions for future research, suggested specifically by this synthesis, include:

1. Rapid and successful adaptation of biological systems, at the cell and tissue level, to *novel* perturbations should be considered with respect to the cognitive task of inferring which genes to activate (microscale) to deal with a physiological stressor (macrostate) (Elgart et al., 2015, 2016; Soen et al., 2015; Schreier et al., 2017). Is there a quantifiable sense in which biological systems model themselves? Can tools such as information theory or other approaches be used to identify signatures in time-series data of regulatory events of attempts by gene-regulatory and bioelectric networks to coarse-grain themselves – to discover their own causal structure for optimally efficient self-control?
2. Can an *in silico* evolutionary system be built, containing both genetic and physiological components, which simulates the scheme described above (homeostasis and infotaxis) and illustrates the emergence of different scales of cognitive horizons over time? Can we directly observe the evolution of multicellular goals from networking of agents with single-cell homeostatic goals?
3. Because of the mechanistic and functional commonalities between cognition and patterning, neurotransmitters are ancient control mechanisms functioning also outside the nervous system in fungi, plants, and animals. It is already known that perturbation of these pathways can induce low-level effects [perturb developmental patterning (Buznikov and Shmukler, 1981; Hernandez-Diaz and Levin, 2014; Sullivan and Levin, 2016)] but might there be higher level effects on the physiological boundaries of the cognitive sub-agents within the body from exposure to psychedelics, which have long been claimed to expand “transpersonal boundaries” or induce “ego death” (Bouso et al., 2015; Carhart-Harris et al., 2016)? Specifically, the implementation of multicellularity by the drive to minimize predictive error (Fields and Levin, 2019) suggests that cells need a way to keep other cells nearby. This suggests the testable hypothesis that the addictive power of opiates and similar molecules derives from such ancient roots. Functional experiments targeting addiction pathways can readily be attempted in early models of multicellularity and in somatic/stem cell co-cultures *in vitro*, to identify or disprove an ancient role for addictive neurochemicals in this process.
4. It would be instructive to start fleshing out the cognitive light cone diagrams with behavioral data from real organisms and available AI agents, as well as single cancer cells *in vitro* and *in vivo*, and tumors. Additional concepts in this space may need to be added, such as conservation principles; e.g., the amount of “mental energy” for caring about things and making decisions is certainly limited in humans (Weippert et al., 2018); can we measure and define the thermodynamic/metabolic costs of decision-making in basal cognition? More broadly, the analogies between geometric spaces and cognitive ones needs to be explored further, as is beginning to be done *via* geometric information theory (Balduzzi and Tononi, 2009; Oizumi et al., 2016; Sengupta et al., 2016; Calcagni, 2018). Indeed, the notion of spaces with different geometry has been pursued both in the neuroscience of perception and representation (Zhou et al., 2018b; Gilead et al., 2019) and in developmental biology of patterning (Jaeger et al., 2008; Jaeger and Monk, 2014). It may even be possible to go backwards, asking what cognitive structure accompanies a particular space-time geometry – a concept that might have been presaged by Newton’s concept of space being the “Sensorium of God”.
5. A major question in AI is motivation (McShea, 2013) – will complex engineered systems set novel goals, and if so, how? It is usually extremely obvious how to negatively motivate a living organism, but how does one punish an artificial agent? It may be that until we have artificial constructs that are, at their core, homeostatic goal-seeking systems, which will be as easy to motivate as living things, significant general AI will not be possible. While biology has competent agents at each scale (McShea, 2012), most human artifacts do not – robots are often made of reliable but very dumb parts [although modular designs with sub-goals have already been shown to be a useful strategy (Cudhea and Brooks, 1986)]. It is a prediction of this view that adaptive, useful robotics will require components that are themselves competent and goal-seeking. The fact that robot cancer is not a known problem today is likely the same reason we do not have highly adaptive, robust robotics: selection of activity (feedback based on positive and negative reinforcement toward local goals) is applied only at one level. Subunits rich enough to make a good cognitive Self will occasionally defect and go off the reservation as human cells sometimes do. This suggests a roadmap for engineering agents that are fundamentally based on homeostatic goals and selfish infotaxis at multiple levels of organization.
6. The importance of physiological connectivity in keeping cells harnessed toward a global body plan and away from cancer has long been known. The lack of multicellular information is a potent carcinogen; e.g., plastic barriers (but not the same plastic powder) placed between cells induces tumors, as do gap junction blockers (Oppenheimer et al., 1953; Bischoff and Bryson, 1964; Yamasaki et al., 1995). Data in amphibians already show that modulation of the bioelectric state and connectivity of cell networks can prevent and reverse tumorigenesis (Chernet et al., 2015, 2016); thus, the next work in this subfield should focus on the discovery of bioelectric communication-inducing technology [whether through gap junctional opener drugs or bioelectric nanomaterials (Jayaram et al., 2017) for cancer reprogramming as an alternative to chemotherapy]. Another possibility is that stress (physiological, or informational – signals that cannot be predicted by a cell) could be a factor that motivates cells to reduce their sensory/action surface and contract, in effect abandoning organismal membership when it begins to generate more stress than its presence reduces by its protective action. The current research on the relationship between stress and cancer (Benndorf and Bielka, 1997) could be modeled from the perspective of surprise minimization.
7. The proposed crucial role of bioelectrics in multicellularity predicts that it should be possible to induce the formation of metazoan-like bodies in an otherwise unicellular organism by forcing the expression of appropriate electrogenic proteins and gap junctions. Similarly, it may be possible to induce dissolution of an entire metazoan body by appropriate changes of bioelectric dynamics.
8. Bioelectrics should be widely conserved in regeneration across Kingdoms; transcriptomic analyses of regeneration events from damaged bacterial biofilms to plants to metazoan models of limb and organ regeneration should all show consistent use of electrogenic proteins in the events that enable cooperation toward self-limiting morphogenetic cascades. Likewise, it should be the case that neurotransmitter signaling (a key downstream response module for bioelectric change) should be highly conserved, revealing effects of cognitive modulator drugs on function in bacterial biofilms and primitive cognition models such as *Physarum* (Maier et al., 2018).
9. The evolution of neural control systems from pre-neural morphogenetic mechanisms suggests that there should be a lot of interplay between these systems in development. The requirement of a functional CNS for effective organ regeneration (Brockes, 1987) and for correct patterning in development (Herrera-Rincon et al., 2017; Herrera-Rincon and Levin, 2018) has been shown in a range of model species, and the tools of optogenetics can now be used to dissect the information content of nerve-mediated signaling as instructive cues for both normal morphogenesis and regulative development in which bodies adapt to major changes in architecture (Oviedo et al., 2010).
10. The continuum view of cognition suggests that the cognitive ability to learn from experience and act to maximize specific parameters to increase welfare is universal across scales. Much work remains to characterize and exploit phenomena such as cardiac memory (Chakravarthy and Ghosh, 1997; Zoghi, 2004), and learning in bone (Turner et al., 2002; Spencer and Genever, 2003) and in gene-regulatory networks (Herrera-Delgado et al., 2018). Emerging technologies for real-time, closed-loop controls (Bugaj et al., 2017; Perkins et al., 2019) now enable interrogation of cellular cognition in a variety of somatic contexts *ex vivo*. We have previously suggested the use of behavior shaping and training paradigms as a strategy for synthetic morphology and regenerative medicine that complements bottom-up rewiring at the molecular level (Pezzulo and Levin, 2015, 2016; Mathews and Levin, 2017).
11. Similarly, it should be possible to communicate with (train) swarm organisms. Moreover, the scale-free cognition hypothesis suggests that multi-human systems could have their own degree of cognition. We speak this way informally (the Supreme Court has opinions, a town may send letters to specific people, and countries are often portrayed as having intentions and behavioral traits in political planning). Tools of information theory and reinforcement learning techniques could be tested on the behavior of human social groups, to determine if there is a true psychology of an integrated Self that can be predicted and managed as a coherent goal-seeking individual. It is fascinating to note that swarm cognition, in the sense of a new supervenient Self which makes choices and reaps the consequences, is a concept proposed long ago in pre-scientific thought about cognition (Hutchinson and Sharp, 2008).
12. Ascertaining the functional set points (goals) of a given system has implications for open questions in exobiology and artificial life. First, it facilitates identification of a system’s boundaries – empirical hypotheses about what subset of a complex biosphere is practical to consider a unified Individual. This is sometimes obvious in the world of terrestrial biology, but it is an important potential problem to be encountered in research in Life as it Could Be (Langton, 1995). Second, it enables communication with highly diverse intelligences (evolved or constructed), with whom we may share no language and few mental constructs. Given a novel life form, how does one know when successful communication has taken place? A basic form of communication is behavior shaping: if a system can be made to act in a pre-determined way through positive and negative reinforcement, then one is sure that at least one thing has been communicated: the desired activity. Of course this is a very minimal notion of communication, but it is a practical and useful starting point in scenarios where the biology or engineering is truly novel. This requires understanding what motivates the system (i.e., what conditions it seeks vs. avoids) and takes advantage of its innate goal-seeking behavior.

## What Does It Feel Like to be a Pancreas?

Most of the prior discussion focused on objective, functional traits, and capabilities and is compatible with several views on consciousness. Nothing in this model explains why specific functional features give rise to first-person experience (the so-called Hard Problem). But, given that at least some nervous systems do give rise to such experience and that the differences between neural networks and non-neural ones (aside from temporal scale) are minimal, it is natural to hypothesize that there is something-it-is-like to being a tissue or organ and making decisions. It is not claimed of course that a pancreas, in its striving to keep homeostasis, has any self-awareness in the human sense, but it may have as much proto-consciousness as a simple neural network and indeed diabetes has already been modeled as a kind of cognitive disorder (Arntfield and van der Kooy, 2011; Goel and Mehta, 2013). The model does take a stand on the perennial “combination problem” and can perhaps be seen as a form of panpsychism (Chalmers, 2013).

One final comment concerns an interesting intersection of the above model with non-Western views on consciousness. It is striking that the process which Zen practice is meant to reverse – attachment to past memories and high valence for future expectations/fears – is precisely the process suggested to be responsible for the creation of complex Selves. It is unclear whether it is beneficial (or even possible) to truly live in the moment and let go of past memories and future expectations, but anyone who succeeded in doing this would achieve precisely what Zen promises: the dissolution of the self (Flanagan, 2011; Josipovic, 2014, 2019). According to the above model, the Zen ideas of stamping out desire (goal-directed activity, preferences for specific states of affairs) are exactly correct in that this would lead to a dissolution of the ego (Self) and the freedom from the law of cause and effect that governs the Individual’s actions. By turning off memory, anticipation, and striving, the essential glue that creates a cognitive Self is dissolved. By erasing the set point toward which the feedback loop expends energy to accomplish, the higher level integrated Self disappears, leaving nothing but the constituent parts (smaller Selves in their own right). This is completely different from killing the individual components, and it can be asked whether someday we will develop a biochemical path to the Nirvanic Void, which releases the global self by breaking the integrating communication channels but leaves all of the subunits healthy and free to pursue their local goals. Re-creating the unification into, and liberation from, larger scale unifying Selves would be a true pinnacle of synthetic biology and artificial intelligence engineering.

## Author Contributions

The author confirms being the sole contributor of this work and has approved it for publication.

### Conflict of Interest

The author declares that the research was conducted in the absence of any commercial or financial relationships that could be construed as a potential conflict of interest.
